# The Sterile Inflammation in the Exacerbation of HBV-Associated Liver Injury

**DOI:** 10.1155/2015/508681

**Published:** 2015-03-29

**Authors:** Qiao Yang, Yu Shi, Ying Yang, Guohua Lou, Zhi Chen

**Affiliations:** ^1^Department of Infectious Diseases, Sir Run Run Shaw Hospital, School of Medicine, Zhejiang University, Hangzhou, Zhejiang 310016, China; ^2^State Key Laboratory for Diagnosis and Treatment of Infectious Diseases, The First Affiliated Hospital, School of Medicine, Zhejiang University, Block 6-A, 17th Floor, No. 79 Qingchun Road, Hangzhou, Zhejiang 310003, China; ^3^Collaborative Innovation Center for Diagnosis and Treatment of Infectious Diseases, Hangzhou, Zhejiang 310003, China

## Abstract

Exacerbation of hepatitis B virus-associated liver injury is characterized by abnormal immune response which not only mobilizes specific antiviral effects but also poses a potentially lethal nonspecific sterile inflammation to the host. How nonspecific sterile inflammation is triggered after the preexisting injury caused by specific immune injury remains elusive. In the setting of sterile inflammation, endogenous damage-associated molecular patterns are released by stressed and dying hepatocytes, which alarm the immune system through their potential pattern recognition receptors and related signaling pathways, orchestrate the influx of diverse cytokines, and ultimately amplify liver destruction. This review highlights current knowledge about the sterile hepatic inflammation in the exacerbation of chronic hepatitis B.

## 1. Introduction

Hepatitis B virus (HBV) infection is a serious health problem because approximately one-third of the world population has been infected with HBV at a certain moment of their lifetimes including more than 350 million people who are chronically infected [1]. Persistent HBV infection may eventually cause progressive complications including cirrhosis, hepatocarcinoma, and liver failure. Most hepatocyte damage caused by HBV infection is attributed to the immunopathological response triggered by viral antigen rather than the direct injury of massive HBV replication [2]. A potent, virus-specific cytotoxic T lymphocyte- (CTL-) response is essential for virus elimination; however, this response is not usually sustained. The impairment of a CTL-response can predispose a latent or persistent viremic infection. A few proportions of patients with chronic HBV infection can develop acute hepatitis flare, called acute-on-chronic liver failure (ACLF) or fulminant hepatitis (FH) [3], which is an extreme phenotype of liver injury. Despite being a relatively uncommon condition, none is as dramatic, devastating, and complex as FH, and this may explain the difficulty in rapid diagnosis and clinical management. So, it is important to identify the mechanisms that predispose the acute exacerbation of chronic hepatitis B virus infection.

## 2. The Path to HBV Infection

There are at least two types of immune responses in patients with HBV infection: the HBV-specific and the non-HBV-specific immune responses.

The HBV-specific immune response is directed and primed by antigen-presenting cells (APCs) in association with viral antigen and the major histocompatibility complex (MHC) molecules. The HBV-specific T helper lymphocytes activate and produce interleukins and interferon proteins, which in turn mobilize CTL to kill the specific pathogen and fulfill antiviral function. In addition, the humoral immune system produces HBV-specific antibodies to defeat the invading antigens. In most adults with acute HBV infection, HBV-specific cellular and humoral immune responses are prompt, vigorous, and polyclonal, which lead to a self-limited disease with lifelong protective immunity. However, the impairment in the immune response predisposed by the increased expressions of membrane inhibitory receptors (e.g., programmed death- (PD-) 1 and T-cell immunoglobulin and mucin domain-containing molecule 3 (Tim-3)) can cause asymptomatic or chronic inflammation in the liver (called inactive carrier state and chronic hepatitis B (CHB), resp.) [4–7]. The nonspecific innate immunity is initiated early and includes the phagocytic cells (e.g., liver sinus endothelial cells (LSECs), monocytes/macrophages), natural killer (NK) cells, dendritic cells (DCs), proteins of complement system, and acute phase proteins. HBV seems to avoid inducing strong innate immune response in the early time of infection [8]. However, the role of the innate immune response in the control of viral replication should not be ignored. For example, the clearance of HBV can be mediated by potent antiviral cytokines (especially type I interferon) that is derived from activated innate immune cells [9, 10].

Approximately 0.2–4% of HBV infection is associated with inflammatory exacerbation through a number of scenarios, such as hyperactive immune response after acute infection, virus mutation, immunosuppressive therapy, immune reconstitution, and superimposition with delta virus on a chronic HBV carrier state [11]. The precise mechanism involved in the exacerbation of HBV infection is not completely understood. Although the destruction of HBV-infected cells is widely thought to be mediated by MHC class I-restricted CD8^+^ CTL through a Fas/FasL cytotoxicity and a perforin/granzyme cell death pathway [12], the effects of CTL are not sustainable. Also, the response evoked by the trafficking and recruitment of innate immune cells fails to explain such a devastating inflammation in CHB flare. There is a significant emerging body of evidence that the non-HBV-specific sterile inflammation triggered by endogenous damage-associated molecular patterns (DAMPs) can worsen preexisting immunopathology and may result in the deterioration of liver inflammation. This review addressed the contribution of sterile inflammation to the exacerbation of HBV-induced liver injury.

## 3. Hepatic Predisposition to Sterile Inflammation

The liver is a highly vascularized organ, which receives dual blood supply from the hepatic arteries and the portal venous system. A large portion of blood (70–80%) comes from the portal system which continuously delivers gut-derived nutrition and microbial metabolites to the liver; the hepatic sinusoids are therefore constantly faced with the external challenges. The hepatic sinusoid is composed of a diversity of immunologically active immune cells, including liver sinusoidal endothelial cells (LSECs), Kupffer cells (KCs), and hepatic stellate cells (HSCs). Additionally, natural killer (NK) cells, natural killer T (NKT) cells, and dendritic cell (DCs) are often present in the sinusoidal lumen [13–16]. The functional states of these “guide” cell types may largely determine whether or not an insult requires mobilization of the immune response. Altogether, this unique anatomical and physiological characteristic of the liver provides a distinctive local immune environment and confers its vigorous ability to respond to dangers.

## 4. The Initiation of the Sterile Inflammation during HBV Infection

Sterile inflammation actually occurs in all tissues after injury of various etiologies. Very little has been known about how non-HBV-specific sterile inflammatory response is triggered after the preexisting injury caused by specific-CTL. The story after hepatocyte dying does not just rely on the virus induction, and the danger signals, also named damage-associated molecular patterns (DAMPs) released by the necrotic hepatocyte itself, may be the most likely inflammatory cues [17]. In addition, the secretion of DAMPs also occurs as a regulated event independent of necrosis. DAMPs could also be actively secreted by immune cells (e.g., monocytes, macrophages, and dendritic cells) in response to inflammatory stimuli [18]. In general, DAMPs released by injured hepatocytes act as “alarmins” in order to “inform/alert” the immune system to potential threats and contribute greatly to the subsequent immune response and liver damage. Researches in the field of sterile inflammation have exploded in the last few years, as evidenced by the rapidly expanding list of DAMPs [19, 20]. Significant advances have been made in the illumination of their potential receptors and the downstream signaling events. With these advances has also come an understanding of the critical roles of DAMPs in the development and progression of HBV-associated liver injury. The following sections will focus on recent advances in the understanding of biological functions, molecular mechanisms of DAMPs, and the interplay between DAMPs and immune cells during HBV-associated liver injury.

### 4.1. Damage-Associated Molecular Patterns

#### 4.1.1. Heat Shock Proteins

Heat shock proteins (HSPs), a set of highly conserved proteins from prokaryotes to eukaryotes, function with other molecular chaperones to mediate the correct protein folding and rectify the misfolded proteins [21]. HSPs may be the most extensively studied DAMP in the context of HBV infection. A number of HSPs (e.g., HSPA8, HSP70, and HSP90) have been reported to be supportive factors in the process of HBV replication, and selective inhibition of these HSPs could be host-based anti-HBV strategies [22–25]. Though normally settled in the cellular protoplasm, HSPs can be passively released by necrotic cells to act as a DAMP to elicit nonspecific immunity. It has been demonstrated that the extracellular HSP70 specifically binds to human monocytes/macrophages and induces cytokine production through the CD14, calcium, and p38 mitogen-activated protein kinase pathway [26, 27]. It has also been reported that glycoprotein 96 (gp96), HSP65, and HSP70 help to augment antigen-specific cytotoxic T lymphocyte response, enhance dendritic cell maturation, and boost antigen presentation capacity of macrophage, which not only prompt HSPs as a target for immunotherapy against HBV-related diseases but also predispose the sterile inflammation from innate immune cells [28–33]. If the HBV cannot be cleared entirely, both the infective and sterile inflammation synergically contributed to the exaggeration of chronic hepatitis.

#### 4.1.2. Uric Acid

Uric acid (UA) is the endogenous oxidation product of purine metabolic pathway. Although UA is soluble in the cytoplasmic region, extracellular UA readily forms bioactive urate crystals [34, 35]. Urate crystals released by dying cells can trigger innate immune cell (e.g., neutrophil) activation through TLRs-inflammasome pathway and perform a proinflammatory DAMP function that helps to stimulate inflammatory response [36]. Higher UA levels have been shown in patients with CHB [37], and the lower ratio of UA to creatinine may be linked to higher mortality rates of fulminant hepatitis [38]. In the study of mice by Kono et al., genetic and pharmacologic manipulation that reduced UA levels specifically inhibited the cell death-induced inflammatory responses [34]. Also, UA has been shown to stimulate dendritic cell (DC) maturation and immunization with DC-plus-UA enhances the HBsAg-specific-CTL cytotoxicity [39]. The above studies prompt us to propose that UA is a promising candidate for both anti-inflammatory and antiviral therapies. However, treatments aiming at anti-HBV with UA adjuvant have to be used carefully, as the sterile inflammation augmented by excessive UA has to be taken into account.

#### 4.1.3. Nucleic Acids

One of the hallmarks of hepatitis is the persistent cellular apoptosis or necrosis to varying degrees. On one hand, the degradation or disruption of nuclear DNA can be released into extracellular compartment, which subsequently results in TLR9-associated proinflammatory transcription factors (e.g., nuclear factor-кB (NF-кB) and activator protein 1 (AP-1)) activation and the production of a number of cytokines (e.g., TNF-*α*, IL-6, and interferon-*α*) [40–42]. On the other hand, the double-strands RNA derived from necrotic cells can stimulate TLR3 through MyD88-dependent or MyD88-independent signaling pathway [43, 44]. Many early studies have suggested that the immune response driven by the endogenous nucleic acids is the key component of sterile inflammation in an array of conditions, such as acetaminophen-induced hepatotoxicity and ischemia-reperfusion liver injury [45–47]. Under the condition of CTL-mediated killing of infected-hepatocytes, the nuclear acid fragments of viral origin and the DNA fragments of dying-hepatocyte origin could collectively amplify the inflammation in the context of HBV infection.

#### 4.1.4. High Mobility Group Box 1

**Figure 1:**
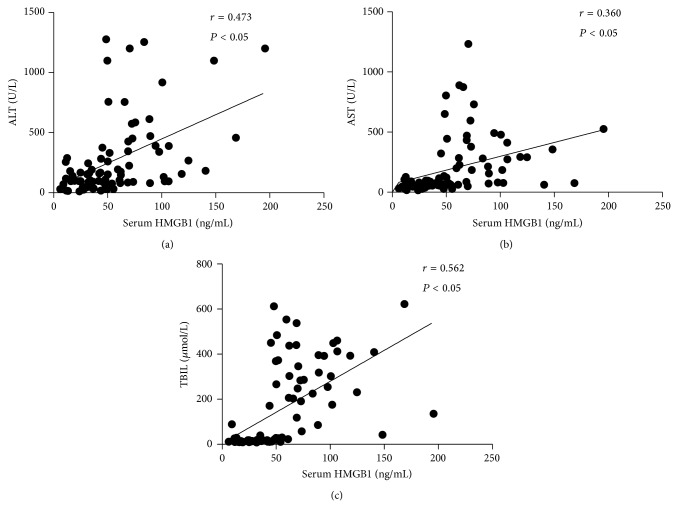
The serum levels of HMGB1 in patients with CHB. (a)-(b) Correlation between the serum HMGB1 levels and ALT or AST in patients with CHB. (c) Correlation between the serum HMGB1 levels and TBIL in patients with CHB. Each dot represents data from one subject. The data were analyzed by the Spearman rank correlation test. HMGB1: high mobility group box 1; ALT: alanine aminotransferase; AST: aspartate aminotransferase. CHB: chronic hepatitis B; TBIL: total bilirubin.

One of the most frequently studied DAMPs relating to sterile inflammation may be high mobility group box 1 (HMGB1), a highly conserved nuclear protein that facilitates the nucleosome stabilization and regulates the transcriptional activation [48, 49]. The biological activity of HMGB1 is much more complex than previously thought. Extracellular HMGB1 from dying cells can act as a “danger signal” to elicit innate and adaptive immune response through a wide range of receptors (RAGE, TLR4, TLR2, and TLR9) [50–53]. A number of experimental reports have linked HMGB1 with HBV-associated liver diseases. Increased levels of HMGB1 in serum and in HBV-infected hepatic tissue as well as the translocation of nuclear HMGB1 have been described in HBV-associated acute liver failure (ALF), and the serum levels of HMGB1 were positively correlated with disease severity in patients with CHB [54–57] (Figure 1). Besides, the pathogenic effects associated with HMGB1 could be ameliorated by antibodies against HMGB1 proteins or exacerbated by administering exogenous recombinant HMGB1 to animals [58–61]. A few experimental data addressed the molecular mechanism of HMGB1 involved in the pathogenesis of HBV infection. A report by Jie and coworkers showed that decreased binding affinity of HMGB1 to the -3 region of IFN-*α*/*β* receptor 1 (*IFNAR1*) promoter results in a decreased IFNAR1 expression, which in turn affects the susceptibility and the clinical outcome of chronic HBV infection [62]. Some authors reported that HMGB1 can impair the immune activity of regulatory T (Treg) cells through suppressing Foxp3 gene expression, indicating another pathogenic mechanism of HMGB1 in ACLF [57]. However, HMGB1 alone is unlikely to lead to an efficient proinflammatory effect; interplay with other DAMPs can trigger potent inflammation [17, 63–65]. For example, as a nuclear DNA binding protein, extracellular HMGB1 combines with circulating self-DNA so as to recruit TLR9 in pDCs via RAGE and further activate TLR9-MyD88 pathway [66, 67]. Also, HMGB1 forms complex with IL-1*β*, greatly potentiating the proinflammatory activity of IL-1*β* alone [65]. Recently, several possible therapeutic approaches, including neutralizing antibodies or pharmacological inhibition of HMGB secretion, have been evaluated in animal models and are currently being translated into preclinical trials [58].

#### 4.1.5. Interleukin-33

IL-33 is a novel member of IL-1 family that is proved to be capable of activating T helper type 2 immune response via IL-1 receptor-related protein ST2 [68–70]. Similar to HMGB1, IL-33 functions as both a secreted cytokine and a nuclear protein with the ability to regulate gene transcription [71]. IL-33 also acts as an alarmin upon tissue injury and as a mechanically responsive cytokine secreted by living cells [72]. The involvement of IL-33/ST2 axis in a number of inflammatory contexts is controversial. To identify the immunopathology of IL-33/ST2 axis, Volarevic et al. genetically engineered mice that are defective of ST2 to test the susceptibility to Concanavalin A-induced hepatitis. This study revealed that mice defective of ST2 showed more severe hepatitis than the wild-type mice with higher number of inflammatory cell infiltration in the liver, and pretreatment of WT mice with IL-33 led to attenuation of the liver injury [73]. Studies conducted on ischemia/reperfusion mice model have indicated the protective effects of IL-33 on hepatocytes, which is associated with the activation of a series of signaling molecules related to cell survival (NF-*κ*B, p38 MAPK, cyclin D1, and Bcl-2) [74]. Other studies suggested that administration of IL-33 protein into wild-type mice has been proven to upregulate the expression of IL-4, IL-5, and IL-13 in the liver which causes Th2-polarized response [75]. These findings highlighted the protective role of IL-33/ST2 axis in liver injury through preventing hepatocyte apoptosis and Th2 amplification.

**Figure 2:**
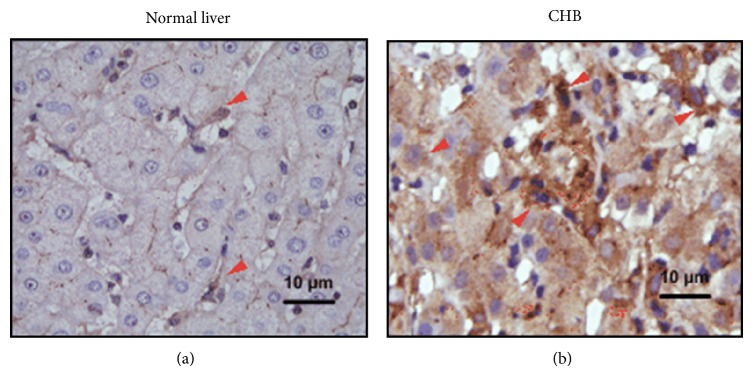
The expression of hepatic IL-33 in patients with chronic HBV infection. Positive staining with IL-33 antibodies is shown in brown. IL-33^+^ cells are mainly located in sinusoids (a) in normal livers but are also detected in inflammatory regions (b) from CHB patients. CHB: chronic hepatitis B; HBV: hepatitis B virus; IL: interleukin.

A recent study reported that the elevated level of IL-33 was observed in patients with liver failure and the IL-33 level correlated with its decoy receptor soluble ST2 level and alanine aminotransferase (ALT) activity [76]. In addition, treatment with antiviral drugs for 12 weeks leads to a significant decrease of serum IL-33 level and recombinant IL-33 protein can suppress the secretion of HBsAg and HBeAg and HBV DNA replication in vitro [77]. Moreover, the immunohistochemistry experiments on the expression of IL-33 in the case of HBV-associated liver injury by our group showed that IL-33 is constitutively expressed in the endothelial cells in normal liver tissue (Figure 2(a)), and increased IL-33 positive staining was observed in immune cells as well as in hepatocytes in the livers from patients with CHB (Figure 2(b)). Thus, it raises the possibility that the IL-33 released by dying hepatocytes during liver injury acts as an alarmin to engage its putative receptor (ST2 receptor) and to trigger further immune response. Yet, the exact molecular mechanism/mechanistic details of the IL-33 in HBV-associated liver injury need to be further investigated.

#### 4.1.6. Other DAMPs

A potential role of DAMPs has recently been established for hyaluronic acid (HA), which can be synthesized and degraded by the liver and has been proposed as a biomarker for high score fibrosis and cirrhosis. Recently, the dysfunction of endothelial cells resulting in the generation of HA has been reported to be important in acetaminophen hepatotoxicity [78]. Therefore, it is worth further studying the role of HA in the sterile inflammation associated with HBV infection.

The cytoplasmic S100 proteins are promising new DAMPs in the pathogenesis of acute and chronic inflammation [79]. S100 proteins are elevated in the serum in chronic viral hepatitis in humans [80] and are a useful marker of hepatic encephalopathy in patients with fulminant hepatitis [81]. It remains to be tested if the release of S100 proteins from an acutely injured liver is responsible for the following damage in HBV infection.

### 4.2. Inflammasomes

Another sterile inflammation relies on a cytoplasmic multiprotein complex termed as the inflammasome, which enables activating protease caspase-1 to regulate the maturation and release of the IL-1 family cytokines (e.g., IL-1*β*, IL-18, and IL-33) in response to exogenous pathogens and endogenous danger signals [82]. The sensors of inflammasome are the NOD-like receptor (NLR) or the HIN-200 receptor family, including Nlp1b, Nlrp3, and Nlrc4, or the absence of melanoma (AIM2). These receptors are recruited into the inflammasome upon recognition of diverse pathogen-associated molecular patterns (PAMPs) and lead to increased transcriptional activity of inflammasome components [83, 84]. A variety of DAMPs are also required for full mobilization of the inflammasome. For example, the internalization of uric acid crystals causes the rupture of phagosomes, which have been identified as potent triggers of NALP3 inflammasome activation [85]. Martinon et al. also reported that the activation of IL-1*β* induced by crystals was attenuated in macrophages deficient in components of NALP3 inflammasome (ASC, NALP3, and caspase-1) [86]. How the inflammasomes sense UA crystals needs to be further characterized. Recently, it has been described that the presence of the purinergic receptor P2X7 and its ligands ATP was also associated with NLRP3 inflammasome activation in acetaminophn-induced hepatotoxicity [87]. In addition, Imaeda and colleagues showed that DNA released from dying hepatocytes has the ability to activate TLR9, which subsequently collaborates with other DAMPs (uric acid or ATP) to activate the NALP3 inflammasome and evoke pro-IL-1*β* and pro-IL-18 cleavage [88]. This study sheds important light on the combined priming signals for the activation of the inflammasome. As to the function of inflammasomes in HBV infection, it has been recently suggested that the activation of purinergic receptor P2X7 is involved in the attachment of HBV to the susceptible hepatocytes and the inflammation in the liver [89, 90]. Therefore, purinergic therapeutic strategies for the treatment of HBV-induced liver damage should be explored. A small clinical study was conducted involving CHB subjects. The major outcome of the study was that HBcAg treatment leads to secretion of bioactive IL-18 in PBMCs in a dose-dependent manner. No suppressive effects were detected when the costimulatory molecule CD40 was blocked. But the caspase-1 inhibitor zYVAD-fmk completely blocked HBcAg-induced IL-18 secretion [91], suggesting the caspase-1 dependency of the pro-IL-18 cleavage in HBcAg-treated PBMCs. Besides, the inflammasome-dependent productions of IL-1*β* and IL-18 are capable of eliciting antiviral response through priming the differentiation of CD4^+^ T cells or evoking IFN-*γ* production [92–94]. These studies not only pave the way to unravel the role of intracellular defense mechanism against HBV by inflammasome activation but also raise the question how the inflammasomes sense the invading HBV core antigen and what are specific signaling pathways initiated by inflammasome complexes in HBV immunopathology. Besides cytokine processing and release, the activation of caspase-1 in inflammasome platform is shown to induce pyroptosis, a specialized cell death pattern that eliminates the infected cells and presents intracellular microbial antigens as part of host defense mechanisms. Further work should identify the involvement of inflammasome-mediated hepatocyte death in HBV infection.

## 5. Innate Immune Cell-Mediated Immunologic Effects after Sensing DAMPs

The initiation of the HBV-infected inflammation results in the infiltration and activation of innate immune cells and the accumulation of different DAMPs from the apoptotic or necrotic hepatocytes. The majority of immunopathological damage in HBV infection is elicited by the nonspecific infiltrating immune cells but not the viral-specific T cells. Besides, the immune response induced by the DAMPs causes collateral damage to the inflamed liver. There is extensive evidence that the signaling events processed by DAMPs are similar to those by PAMPs, and they share the same pattern recognition receptors (PRRs) and related signaling pathways [95, 96]. Thus, DAMPs have similar functions as PAMPs in terms of their ability to provoke inflammation. The following section summarizes current understanding of the potential mechanisms by which the DAMPs modulated the innate immune system and their implications for CHB exacerbation.

### 5.1. Dendritic Cells

Dendritic cell (DC), a member of mononuclear phagocyte lineages, is essential component in bridging innate and adaptive immune response. Both the decrease in circulating DC numbers and the deficits in DC functions are responsible for HBV persistence [97–99]. Given their central role in type I IFN production and regulation of antiviral T-cell responses, the DC-based therapy represents a powerful strategy for treatment of HBV infection [100]. In the chronic inflammatory liver, it is likely that DCs are confronted with DAMPs from surrounding damaged cells and the complex interplay of DAMPs on DCs may affect the ultimate clinical outcome of CHB. For example, the HSP gp96 has been reported to assist in inducing the maturation of human DCs through upregulating the MHC class II and the costimulatory molecular B7-2 [101, 102]. Another danger factor, HMGB1, has been described to play dual roles in DC activation and recruitment, since treatment with HMGB1 led to an increased expression of surface markers (CD80 and CD86) and enhanced production of cytokines (IL-6, IL12p7, and TNF-*α*), and blocking antibody against HMGB1 or its receptor RAGE strongly inhibited the migration of DC in response to chemokines CCL19 and CXCL12 [103, 104]. In contrast, data from Popovic et al. showed that HMGB1 prevented a TLR9 agonist-induced proinflammatory response of plasmacytoid DCs (PDC) and potently inhibited the upregulation of costimulatory molecules on the PDC. This in turn resulted in a downregulation of their ability to cytokine secretion and maturation [105]. The divergent findings may be due to the differences in DC subtype. Endogenous DNA, which is thought to possess specific immunogenic properties, is also able to convey the proinflammatory messages through TLR9 on DC. Ligation of the TLR9 gives rise to MyD88 recruitment, which subsequently activates the NF-*κ*B and AP-1, thus intensifying the inflammatory response through DC activation [106, 107].

### 5.2. Macrophages

Macrophages show a strong predisposition of the innate immune response in the liver and constitute up to 80–90% of the total macrophage population of the body. In the presence of type I cytokines (e.g., IFN-*γ*, TNF-*α*) or PAMPs (e.g., LPS, dsRNA), classical M1 macrophage activation occurs with enhanced cytotoxic activity, whereas, in the case of type II cytokines (e.g., IL-4, IL-13), alternative M2 macrophage activation associates with immunoregulatory and phagocytic effects [108, 109]. Macrophages, as phagocytic mononuclear cells, constitutely express scavenger receptors that mediate phagocytosis of the apoptotic hepatocytes and carry specific danger receptors that sense the DAMPs. Evidence has been presented that KC is of vital importance in viral clearance and maintenance of the course of chronic infection [110, 111]. Nevertheless, it is likely that, in the exacerbating stage of HBV infection, the extensive apoptotic hepatocytes cannot be cleared up immediately, necrotic hepatocytes inevitably occur and release array of DAMPs, which subsequently promotes inflammatory infiltration [110]. Macrophages are therefore exposed to the danger DAMPs and transmit the danger signals in the inflamed tissue. For example, macrophages recognize extracellular UA through TLR2 and TLR4 and promote caspase-1 activation and the release of IL-1*β* Besides, our recent study found that the endogenous HMGB1 acts as an “early alarmin” of macrophage activation through MAP kinase signaling pathwayduring Con A-induced acute liver failure [113]. IL-33/ST2 has been implicated in promoting efficient inflammation by amplifying alternatively activated macrophages polarization and chemokine production [114]. Therefore, the DAMPs may synergically enable the activation of macrophages, thus initiating the inflammatory exacerbation.

### 5.3. Neutrophils

Inflammatory neutrophils represent the most potent effectors of the innate immune system, in that they are responsible for pathogen elimination and the regulation of inflammation through the release of cytotoxic ROS/RNS and proteolytic enzymes [115]. In addition, the apoptosis of extravasated neutrophils induced by pathogen acts as a homeostatic mechanism in inflammation [116]. Previous study has implicated the role of PreS1 fragment of HBV antigen in priming the oxidative burst response of neutrophils [117], suggesting the important role of the nonspecific immune injury in HBV infection. A study by Sitia et al. showed that the exhaustion of neutrophils could alleviate liver injury by blocking the recruitment of nonspecific cells and maintain the antiviral effects of HBV-specific cytotoxic T lymphocytes (CTLs), indicating a potential immunotherapeutic approach for the treatment of HBV infection [118]. In addition to HBV insults, inappropriate necrosis of hepatocytes can induce profound neutrophilic inflammation or guide neutrophil infiltration by releasing the alarmins, which is the critical source of collateral damage during the exacerbation of liver injury [110, 119].

## 6. Adaptive Immune Cell-Mediated Immunologic Effects after Sensing DAMPs

During the progression of CHB, the contribution of adaptive immunity is unique in that the HBsAg-specific CTL executes a noncytolytic antiviral mechanism mediated by interferon-*γ* production and that it also induces an immunopathological injury by Fas ligand and perforin-induced death pathways in hepatic tissue [120]. In addition, the HBsAg-specific CD4^+^ T cells assist B cells to elicit protective humoral immunity to HBV [121]. Alternatively, unconventional T cells, such as Treg cells and Th 17 cells, are also involved in the progression in HBV-infected patients [122–126]. Actually, T cells are simultaneously positioned between the foreign HBV and the endogenous damaged molecules during the deterioration of liver disease, performing dual immunologic functions.

The presence of HSPs in human serum in response to viral infection has been reported to exert a variety of extracellular immunomodulatory functions [127–130]. Experiments by Wachstein J and coworkers have shown that treatment with HSP70 strongly enhances the suppressive functions of Treg cells as measured by the production of IL-10 and TGF-*β* [131]. Also, soluble HSP60 derived from HBV-infected hepatocytes promote the function of HBcAg-specific IL-10-secreting Treg cells, which implies the mechanism involved in the aggregation of HBV infection [129]. Another example concerning the potential effects of the “danger” molecular on the adaptive immunity during CHB progression is that the extracellular HMGB1 depresses the immune activity of Treg cells by inhibiting the expression of Foxp3, thereby amplifying the immune response [57], but how the circulating HMGB1 is scavenged and what are the priming signals in Treg cells are as yet uncertain. In contrast to HMGB1, IL-33 has been shown to promote Th2-polarized humoral antibody response by secreting IL-4, IL-5, and IL-13, which help to recover from infection with HBV by blocking the spread of virus and facilitating the clearance of circulating viral particles [75, 132]. Furthermore, it has been proposed that uric acids originating in necrotic cells accumulate in local microenvironment and lead to Th17 polarization in a NLRP3-IL-1/IL-18 dependent way and both the “alamin” and NF-*κ*B priming signals collaboratively induce DCs to produce Th17 polarizing cytokines IL-1*α*/*β*, IL-6, and IL-23, thus further intensifying the adaptive immune response [133]. Therefore, apart from their exacerbating effects on inflammation, the DAMPs are crucial factors engaged in antiviral immune response.

## 7. Biological Therapeutic Prospects

Despite significant therapeutic advances in the management of HBV infection, specific and efficacious therapies targeting the process of the exacerbation of HBV-associated liver injury are still lacking. Many studies on animal models have taught us much about the course of CHB exacerbation and identified some targets for potential therapies. For example, injection of recombinant IL-33 attenuates hepatic injury by recruiting T-regulatory and IL-4-producing CD4^+^ cells in a mouse model of Con A-hepatitis [73]. Functional blockade of HMGB1 reduces hepatic enzymes and ameliorates liver pathology through lessening the recruitment of polymorphonuclear neutrophils (PMNs) in HBV transgenic mice with CTL-induced hepatitis [59, 62]. So identifying the inhibitors of other DAMPs involved in regulating the inflammatory response to hepatic injury seems highly promising for clinical use [73–75, 78, 80, 89, 90]. Nevertheless, using the experience of animal models as tools to explore approaches to clinical therapy has its limitation. If we are to explore therapeutic potentials in predicting and targeting the progressive inflammation during HBV infection, further investigation of human subjects is clearly needed.

## 8. Concluding Remarks

**Figure 3:**
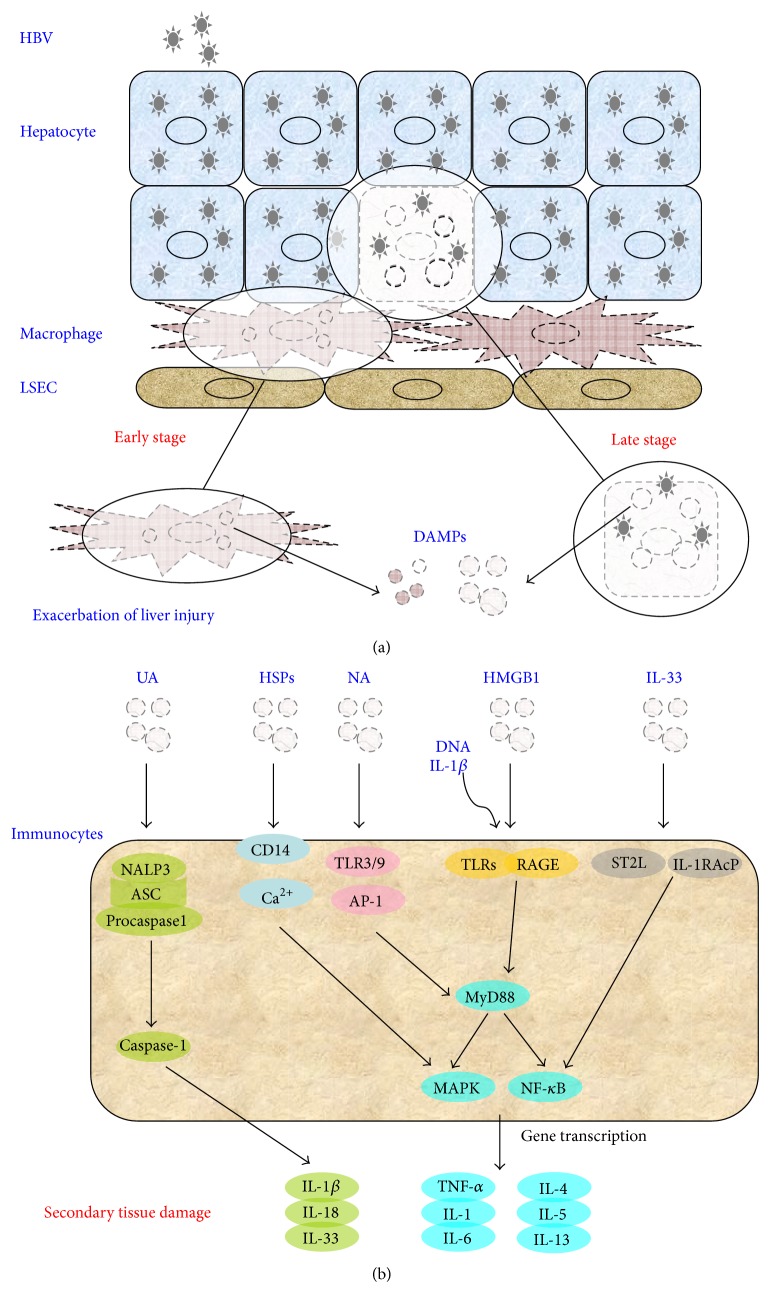
The profile of DAMP-mediated danger signaling in the exacerbation of HBV-associated liver injury. The figure illustrates the self-derived DAMPs which act as “early alarmin” of innate immunocytes in the early stage of hepatitis and the passive released DAMPs which function as late mediator in the process of liver inflammation. AP-1: activator protein-1; ASC, CD: cluster of differentiation; DAMP: danger-associated molecular patterns; DNA: deoxyribonucleic acid; HBV: hepatitis B virus; HMGB1: high mobility group box 1; HSP: heat shock protein; IL: interleukin; LSEC: liver sinusoids endothelial cell; MAPK: mitogen-activated protein kinase; NA: nuclear acid; NALP; NF-кB; RAGE; TLR: toll-like receptor; TNF: tumor necrosis factor; UA: uric acid.

Sterile inflammation has been recognized as being critical to the acute exacerbation of CHB (Figure 3, Table 1). The emerging concept of DAMP signaling is clearly an evolving field in sterile inflammation. The biological characterizations of the DAMPs and their intricate network with the innate immunity indicate that these factors are likely to be pathologically relevant and ultimately clinically important. In addition, it is now clear that various T-cell subsets, such as Treg cells, Th17 cells, and Th2 cells, and inflammasome also participate in sterile inflammation during CHB exacerbation.

Despite a considerable body of researches over the last few years, there are several issues that need further study. Which DAMP is the instigator of sterile inflammation? How is the immune system capable of discriminating between the endogenous DAMPs and liver itself and launching response? What are the factors that handle the immune response to clear HBV without causing excessive liver damage? Besides causing excessive liver damage, does any DAMP initiate tissue repair? Improved understanding of the pathogenesis of CHB exacerbation will provide valid candidate therapeutic targets for severe hepatitis B clinical intervention.

## Figures and Tables

**Table 1:** The effects of damage-associated molecular patterns in HBV-related liver injury.

DAMPs	Effects in HBV-related liver injury	References
HSP	To support the process of HBV replication Induction of nonspecific immunity Upregulation of antigen-specific cytotoxic T lymphocyte response	[21–32]

UA	To trigger innate immunity To act as adjuvants to enhance the HBsAg-specific-CTL cytotoxicity	[34, 38]

NA	Stimulation of TLR-associated signaling pathways To induce an array of proinflammatory cytokines	[39–43]

HMGB1	To elicit innate and adaptive immune response To impair the immune regulatory effect of Treg cells Collaboration with other DAMPs to potentiate inflammation	[48, 50–52] [17, 54, 62–66]

IL-33	Upregulation of Th2-polarized response To protect the survival of hepatocytes Suppress the secretion of HBsAg and HBeAg and HBV DNA replication in vitro	[73, 74, 76]

CTL: cytotoxic T lymphocyte; DAMP: damage-associated molecular pattern; DNA: deoxyribonucleic acid; HBeAg: hepatitis B e-antigen; HBsAg: hepatitis B surface antigen; HBV: hepatitis B virus; HMGB-1: high mobility group box 1; HSP: heat shock protein; NA: nucleic acid; Treg: T regulatory; UA, uric acid.
